# Development and evaluation of deep learning algorithms for assessment of acute burns and the need for surgery

**DOI:** 10.1038/s41598-023-28164-4

**Published:** 2023-01-31

**Authors:** Constance Boissin, Lucie Laflamme, Jian Fransén, Mikael Lundin, Fredrik Huss, Lee Wallis, Nikki Allorto, Johan Lundin

**Affiliations:** 1 Department of Global Public Health, Karolinska Institutet, Stockholm, Sweden; 2 Department of Medical Epidemiology and Biostatistics, Karolinska Institutet, Stockholm, Sweden; 3 Institute for Social and Health Sciences, University of South Africa, Johannesburg, South Africa; 4 Department of Plastic and Maxillofacial Surgery, Burn Center, Uppsala University Hospital, Uppsala, Sweden; 5 Department of Surgical Sciences, Plastic Surgery, Uppsala University, Uppsala, Sweden; 6 Institute for Molecular Medicine Finland FIMM, Helsinki Institute for Life Science HiLIFE, University of Helsinki, Helsinki, Finland; 7 Division of Emergency Medicine, Faculty of Medicine and Health Sciences, Stellenbosch University, Bellville, South Africa; 8 Division of Emergency Medicine, University of Cape Town, Cape Town, South Africa; 9 Pietermaritzburg Burn Service, Department of General Surgery, University of Kwa-Zulu Natal, Pietermaritzburg, South Africa

**Keywords:** Trauma, Diagnosis

## Abstract

Assessment of burn extent and depth are critical and require very specialized diagnosis. Automated image-based algorithms could assist in performing wound detection and classification. We aimed to develop two deep-learning algorithms that respectively identify burns, and classify whether they require surgery. An additional aim assessed the performances in different Fitzpatrick skin types. Annotated burn (n = 1105) and background (n = 536) images were collected. Using a commercially available platform for deep learning algorithms, two models were trained and validated on 70% of the images and tested on the remaining 30%. Accuracy was measured for each image using the percentage of wound area correctly identified and F1 scores for the wound identifier; and area under the receiver operating characteristic (AUC) curve, sensitivity, and specificity for the wound classifier. The wound identifier algorithm detected an average of 87.2% of the wound areas accurately in the test set. For the wound classifier algorithm, the AUC was 0.885. The wound identifier algorithm was more accurate in patients with darker skin types; the wound classifier was more accurate in patients with lighter skin types. To conclude, image-based algorithms can support the assessment of acute burns with relatively good accuracy although larger and different datasets are needed.

## Introduction

Burns are defined as the destruction of tissues (skin or other organs) due to energy transfer caused by heat, friction, cold, radiation, electricity or chemicals^1^. They are a common injury of daily life globally with an estimated number of 153,000 deaths annually globally^2^, and a much larger number of non-fatal cases. Not only are they common, they also often affect the most vulnerable parts of populations such as children, elderly, those with poor living conditions, low income or immigrant status residence^3^. Correctly assessing the extent and depth of an acute burn is difficult and misdiagnosis has large consequences for both the patients and the burn centres^4,5^. Indeed, there is evidence that visual assessment of burn depth at bedside by front line clinicians^6,7^ and surgeons^7,8^ can be inaccurate^6^ with errors occurring as often as in 25–39% of cases. Distinguishing early those burns that will heal spontaneously and can be managed conservatively (often at a lower level of care) from those that require surgical intervention (e.g. excision and skin grafts) is critical not only to reduce the risk of over- and undertreatment of the patient, but also to not overwhelm scarce specialized resources^9^.

The most equipped burns centres have access to technologies like laser doppler or optical coherence tomography to enhance their performances in order to diagnose depth and estimate the need for surgery^10^. Such equipment is however very expensive and uncommon in both high-income^11^ and low-income settings^12^. Besides being ill equipped, burns centres in low-income settings struggle also with high caseloads and under-resourced emergency care services^13,14^. Indeed, in those settings it is not only the equipment that is lacking, it is also the number of specialists who can provide accurate diagnosis for all those who need it^15^.

For front line clinicians at the point of care it is critical that they receive timely diagnostic assistance from burns experts in order to improve patient outcome and perform triage. Remote consultation through mobile health (mHealth) applications which are now deployed in many clinical environments is a potential solution^16^. Studies reveal that they both provide front-line clinicians with accurate diagnosis for burn depth^17^ and extent^18^ and that they receive acceptance at both ends, i.e. from front-line clinicians and burns specialists^19^.

A step further from remote image-based diagnostic assistance given by burns experts is through automated processes, as was suggested in recent studies^20^. Several automated procedures for burn depth estimation have been suggested with different levels of technological sophistication^21–33^. At an early stage, hand-crafted image features from a small number of images were used with the aim to segment the burn with the help of the user, and then classify the burn depth into three or four categories^24,25^. More recent approaches take advantage of deep-learning methods such as convolutional neural networks (CNNs) with transfer learning to differentiate burn areas from either normal skin^21–23^ or other types of wounds^26,27^. Regarding burn depth classification, the reported deep-learning algorithms resulted in accuracies between 81 and 95% when classifying depth, including healthy skin^28,30^. These studies however often use images collected through online searches and lack appropriate burn diagnosis^30–33^. Indeed, through a systematic review, we have identified that despite the increased accuracy in burn automated diagnosis, especially for burn segmentation, there are currently large risks of bias in studies at hand, and improved results are needed^20^. Another limitation is the fact that most studies were based on images from fair types of skin. This has implications severe for training of algorithms which will likely not be representative of all populations. It is also a big limitation from a clinical perspective as most cases occur in the most vulnerable populations, including in South-East Asia and Sub-Saharan Africa, who could benefit the most from this types of technologies^34^. In fact, only one study has included burns from Caucasian and African patients and demonstrated the complexity of training an algorithm in a mixed skin types environment^21^.

Given the current evidence for the development of automated burn diagnosis, but the lack of appropriate training material, especially including patients with different skin types, this study was embarked with an aim that was three-fold: to develop and assess image-based deep-learning algorithms that identify burn wounds, classify them based on their depth whether they need surgery (such as skin grafting) or not, and assess accuracy of the algorithms among several skin types.

## Methods

Hereafter we will present the methods used for database generation, annotation, model development and statistical analyses.

### Image database and annotations

A database of images has been assembled and is composed of two separate datasets in order to represent patients with different skin colour (also known as Fitzpatrick skin types^35^). There are six Fitzpatrick skin prototypes, which are a constitutional characteristic of the patient from birth, and which characterize the colour of the skin as well as its reaction to ultraviolet radiation exposure^35^. Our two datasets consist of one collected in Sweden and including mostly patients of North-Germanic origin with lighter skin types (Fitzpatrick skin types 1–2). The second was collected in South Africa and includes mostly patients of South African origins with mixed and darker skin types (Fitzpatrick skin types 3–6).

The Swedish cases were all collected at the burn centre at Uppsala University Hospital between 2006 and 2019. Pictures were taken as part of routine care from arrival to discharge of the patient in order to follow up on wound progression. Patients were then contacted in 2018 to obtain informed consent of these images for the current study.

The South African cases were collected from 2016 to 2018 at one of three burns centres in the country: Tygerberg Hospital and Red Cross Children’s War Memorial Hospital in Cape Town, Western Cape Province, and Edendale Hospital in Pietermaritzburg in Kwa-Zulu Natal. Referrals to these burns centres through the burn section of an image-based local mHealth App: Vula Mobile (www.vulamobile.com) submitted between 2017 and 2018 were also included. Consent was obtained at point of care as part of routine care.

All images and associated information were pseudonymized and stored on a secure server (ownCloud, ownCloud Inc, Lexington, MA) managed by Karolinska Institutet and located in Stockholm, Sweden. Patient information included age group (children and adults) and sex, injury information included body part involved, burn mechanism, and burn depth. Burn depth was assessed by a burn expert as part of the routine clinical evaluation, either at bedside on arrival of the patient to the respective burns centre, or by a burn expert remotely when giving an image-based remote diagnosis based on images through the Vula Mobile app^17^

In order to be included, images had to present acute burns (photographed within 48 h post injury, which is defined as the period of shock^36^). In order to mimic most appropriately the clinical settings where only clean, scrubbed wounds are accepted for remote consultation, wounds included had to be undressed, cleaned and scrubbed (all blisters removed) prior to the picture being taken. All pictures not fulfilling those criteria were excluded.

The final database comprised of 391 (35%) images collected in Sweden and 714 (65%) of images collected in South Africa. A total of 387 patients were included, of which 198 (51%) were children. This resulted in 1105 images representing various body parts of which 339 (31%) required surgery.

To improve the discrimination between the burn wound, normal skin and background such as clothes, bed sheets and various objects in the surrounding, a number of background images were obtained from two publicly available online datasets^37,38^. Images were specifically selected to contain some human body parts with skin present, and with a diversity of skin types.

To mimic “real-life” setting, no standardization protocols were provided for image capture. Indeed, it is believed that if there were to be such protocols in some settings, point-of-care clinicians would not provide images for remote consultation. Images were therefore collected with varying background, devices used, distance from the wound, orientation, flash use, or size. Using previously defined anthropomorphic measurements^39,40^, the pixel size of all images was approximated and set individually on the algorithm training platform (Aiforia Hub, Aiforia Technologies, Helsinki, Finland) in order to adjust the scale of the photographed body parts and the variable range of age groups.

All images were individually and manually annotated to segment the burn wound from normal skin (or background) on a pixel-by-pixel level using binary masks created with an image annotation software (ImageJ, NIH, Bethesda, MD), or directly on the algorithm training platform (Aiforia Create, Aiforia Technologies, Helsinki, Finland). Annotations done using ImageJ were programmatically imported to the training platform. Annotations were performed by trained nurses and medical students familiar with burn injuries under the supervision of CB (South Africa) and JF (Sweden). Several images were annotated by at least two annotators and verified through close collaboration with burn experts. Regarding the surgical classification annotations which requires extreme levels of competences in the field, these were made on an image-level based on the burn’s expert depth diagnosis which is considered gold standard. Previous work using some thirty images also included in this study has shown acceptable agreement in over two thirds of the images^17^.

### Training of the deep learning algorithms

Considering the latest development of research in the field, as well as the complexity of the wound patterns to be classified, we assumed that a probabilistic model would be the best approach, we therefore decided to train CNNs using a commercially available software (Aiforia Create, Aiforia Technologies, Helsinki, Finland). Two independent and separate models were developed. The first algorithm identified and segmented the burn wounds from the background (everything in the image that is not a burn wound). The second algorithm classified each burn area based on their depth into one of two categories: surgical burns which require surgical intervention, for example for skin grafting, because they are of deep-partial or full thickness; or non-surgical burns which are superficial and superficial partial thickness burns and are manageable with conservative treatment possibly at lower levels of care. Keeping in proportions of images from each skin type and for each severity status, the images were split into two sets: one training and validation dataset consisting 70% (n = 773) of all the images, and a set-aside test set with the remaining 30% (n = 332 images) (see Fig. S1).

For the wound identification algorithm, the training area included the whole image and the burn was the area to be segmented. The 536 background images were only added for training (none of the images were included in the test set) and had no annotations. These images were not used with the aim of specifically recognizing other objects, but rather with the only aim to improve the algorithm for burn segmentation. The hypothesis behind this aim is that this type of algorithm would only be used in the context of an acute burn, rather than for other purposes. For the second algorithm of wound classification, only the wound areas were used in the training, separated into two categories based on the burn depth, either surgery needed or no surgery needed. For both algorithms, the training was performed three times using a random selection of 70% of the images directly by the software, and the remaining 30% was used as a validation set for evaluation of the results obtained as well as for hyperparameter selection. After the verification that all three trainings obtained similar results, a last training was performed using the same settings and 100% of the training set prior to be predicted on the set-aside test set.

In addition to the training and testing performed on the complete dataset, separate trainings with the same settings (see Table 1) and subsequent testing were performed on the datasets separated according to skin type (Fitzpatrick skin types 1–2 vs 3–6).

**Table 1:** Parameter settings of trained algorithms.

Hyperparameter/augmentation parameter	Setting
Iterations	30,000
Weight decay	0.0001
Mini-batch size	20
Mini-batch per iteration	20
Iterations without progress	750
Initial learning rate	0.15
Scale variation	± 40%
Aspect ratio	± 30%
Shear distortion	± 30%
Luminance	± 40%
Contrast	± 40%
White balance	± 5%
Image compression quality	40–60%
Rotation	0°–360°

For all algorithms, hyperparameters are presented in Table 1. Feature size was predefined at 125 units for the wound detection algorithms, and 190 units for the surgery classification algorithms. A unit corresponds to an approximated pixel size following manual adjustment according to anthropomorphic measurements as described above.

### Statistical analyses

Statistical analyses were all performed using Stata for Mac version 15. Average measures with standard deviations were calculated for the results of all three folds of training and validation sets. Wound identification was measured on a pixel-by-pixel level for each image and aggregated measures for all images were used. This assessment was made using sensitivity (percentage of pixels identified as burn areas out of the whole burn area), precision (percentage of pixels of burnt area out of all those identified as a burn by the algorithm), and F1 score (the harmonic mean of the sensitivity and the precision). For background images, the percentage of images in which a burn area was identified was recorded as well as the number of images in which the identified burn area represented more than 5% of the image itself. Analyses for the two stratified trainings and testing by skin type of the patients were performed and statistical difference in sensitivity was measured using a non-parametric Mann–Whitney U-test.

For burn surgical classification, while the outcome is binary (surgical burns of deep-partial or full thickness and non-surgical burns of superficial and superficial-partial thickness) the algorithm defines for each image an area in pixels that would require surgery or not. Receiver Operating Characteristic (ROC) curve as well as area under the ROC curve (AUC) were measured. Images were then classified as a surgical burn when ≥ 1% of the wound’s pixels were identified as such. The success rate was the number of images correctly classified over the total number of images in a given set. Sensitivity represents the percentage of images which had ≥ 1% of pixels identified as surgical burn out of all images that required surgery. Specificity was measured as the number of images which had < 1% of identified surgical burn pixels out of all images that did not require surgery. This was measured first for all training and validation sets, and then in the set-aside test set overall; and for the set stratified by skin type of the patients. Sensitivity and specificity are presented with 95% confidence intervals (C.I.) measured using the Clopper–Pearson (exact) estimation.

### Ethical approval

Ethical approval was granted by the Regional Ethics Board in Uppsala (Dnr 2016/279) for the Swedish part of the study, and by both the Stellenbosch Health Research Ethics Committee (N13/02/024) and the University of Kwa-Zulu Natal Biomedical Research Ethics Committee (BCA106/14) for the South African part of the study. All methods were carried out in accordance with relevant guidelines and regulations.

## Results

The following section will present the results obtained for both algorithms: first that of burn area identification, and secondly of burn severity classification.

### Burn area identification and segmentation

In the three-fold trainings (where three training and validation runs were performed using a random 70% vs 30% of the images), a burn area was identified in 13.1% of the images which did not contain any burns in the training set, whereas 0.5% of images with a burn were not identified as such. In the non-burn images, the area identified was larger than 5% of the images’ total pixels in only 6 out of 1147 images. In the validation set, a burn area was identified in 20.0% of the non-burn images, however this area was larger than 5% of the images’ pixels in only 6 out of the 464 background images. Table 2 presents the results in terms of sensitivity and specificity for the number of images in which a burn area was identified for both burn and non-burn images.

**Table 2:** Success rate, sensitivity and specificity of the number of images where a burn area was identified with burn and non-burn images (as defined as ≥ 1%) in the three-fold training and validation sets.

	Sample size^a^	Success rate (%)	Sensitivity (%)	Specificity (%)
Training set	2751	94.3	99.5	86.9
Validation set	1180	91.4	98.9	80.0

^a^Sample size included images (both burn and non-burn) present in all three-fold trainings and is therefore bigger than the number of images.

Figure S3 presents the results for each of the training and validation sets in the three-fold training. Table 3 presents the accuracy measures for the pooled three-fold training and validation sets as well as for the final training and test datasets. The burn identification algorithm could identify 92.5% and 85.1% of the burn area across all three-fold training and testing sets respectively. In the final training, the sensitivity was of 93.2% while in the test set it was of 86.9%. Figure S2 illustrates graphically the results of the burn identification algorithm.

**Table 3:** Average and standard deviation (SD) sensitivity, precision, and F1 score (in percentage) of the burn area identification algorithm across all three-fold trainings and validation sets as well as for the final training and for the independent set-aside test set using the full dataset.

	Number of images	Sensitivity (%)		Precision (%)		F1 score (%)	
		Mean (95% CI)	SD	Mean (95% CI)	SD	Mean (95% CI)	SD
Training sets^a^	1604	92.5 (91.9;93.1)	12.7	89.6 (89.1;90.1)	10.4	90.6 (90.1;91.2)	11.5
Validation sets^a^	715	85.1 (83.7;86.5)	18.9	82.4 (81.0;83.8)	19.2	81.9 (80.6;83.3)	18.4
Final training set	773	93.2 (92.4;94.0)	11.5	88.0 (87.2;88.8)	11.5	90.2 (89.4;91.0)	11.3
Set-aside test set	332	86.9 (84.9;89.0)	19.1	83.4 (81.5;85.2)	17.3	82.9 (80.9;84.9)	18.7

*SD* Standard deviation.

^a^Average across all threefold training and validation sets; only burn images included.

When the results were split by skin types of the patients, the average sensitivity was higher in cases with a darker skin type than in those with a lighter skin type (Mann–Whitney U test; *P* < 0.001; Table 4).

**Table 4:** Sensitivity, precision, F1 score, and Mann–Whitney U-test for images in the test set which was trained by skin type.

Skin types of the patients	Number of images	Sensitivity (%)		Precision (%)		F1 score (%)		Mann–Whitney U-test
		Mean (95% CI)	SD	Mean (95% CI)	SD	Mean (95% CI)	SD	
Lighter skin (skin types 1–2)	118	78.6 (74.0;83.1)	24.9	81.0 (77.4;84.7)	20.0	76.9 (72.9;80.9)	21.9	*P* < .001
Darker skin (skin types 3–6)	214	89.3 (87.2;91.5)	16.0	88.1 (86.2;89.9)	13.7	87.8 (85.8;89.7)	14.8	

*SD* Standard deviation.

### Classification of burn depth according to the need of surgery

**Figure 1:**
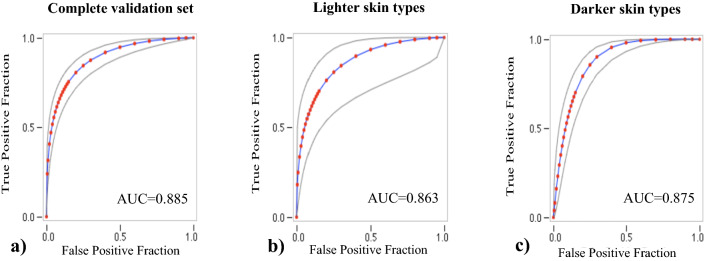
Receiver Operating Characteristic Curve. Wound classifier algorithm (surgery needed versus no surgery needed). (**a**) Combined model. (**b**) Model with training and testing only on lighter skin types. (**c**) Model with training and testing only on darker skin types.

Across the three training folds, almost all the burns requiring skin grafting were identified as such, with a sensitivity of 98%. The specificity was also high with 88%, overall. In the validation sets, the sensitivity was 96% and the specificity was 71%. In the last training, sensitivity was of 99.6% and specificity was of 93.4%. The ROC curves for the set-aside test set as a whole as well as for the two separate skin types are presented in Fig. 1. The areas under the curves were of 0.885, 0.863 and 0.875 in the complete set, and in the images of lighter and darker skin types respectively.

Table 5 presents the summary accuracy statistics for the surgery classification algorithms. The success rate overall was 64.7%, and 78.0% and 66.8% respectively in patients of lighter and darker skin types. The overall sensitivity was 92.5% and the specificity was 53.8%. Indeed, out of the 93 burns which required surgery, only 7 were misdiagnosed. On the other hand, 128 out of 238 burns which did not require surgery were diagnosed as such by the algorithm.

**Table 5:** Results obtained on an image level from the classifying algorithm as compared to the expert’s diagnosis.

	True positive	True negative	False positive	False negative	Success rate	Sensitivity	Specificity	AUC
	n (%)	n (%)	n (%)	n (%)	%	% (95% CI)	% (95% CI)	
Set-aside test set (n = 332)	86 (26)	128 (39)	111 (33)	7 (2)	64.5	92.5 (89.1; 95.1)	53.6 (48.1; 59.1)	0.885
Lighter skin types (n = 118)	15 (13)	77 (65)	21 (18)	5 (4)	78.0	75.0 (66.2; 82.5)	78.6 (70.1; 85.6)	0.863
Darker skin types (n = 214)	71 (33)	72 (34)	69 (32)	2 (1)	66.8	97.3 (94.1; 99.0)	51.1 (44.2; 58.0)	0.875

Average success rate, sensitivity, and specificity (in percentages with 95% confidence intervals), and area under the receiver operating characteristic curve (AUC) for the set-aside test set with all images, and the separate test sets by skin types.

*AUC* Area under the receiver operating characteristic curve.

## Discussion

This study, designed as a proof-of-concept to assist in the diagnosis of extremely complex real-world medical images, evaluates a deep-learning approach for burn wound identification and classification in patients with a full range of skin types. Our results indicate that burns can be identified and segmented with a reasonable accuracy in highly variable pictures captured in real-life settings, whereas classification according to the need of surgery is more challenging. While the burn identification algorithm showed better performance for patients with darker Fitzpatrick skin types, the algorithm for the classification of surgery was less accurate for those patients compared to patients of lighter skin types. We believe the differences in results obtained between skin types and algorithms could be explained by the fact that the difference in colour between burn and normal skin is probably larger for patients of darker-skin types and therefore the wound identifier algorithm performing better in that dataset. On the contrary, the wound classifier performed better in the lighter skin-types which could partly be explained by a higher quality of the images and less diverse image capture settings.

The aggregated results of the accuracy of the wound identification are in line with previous deep-learning studies, as reflected in an F1 score of 83.0%^22,23,41^. Requesting the front-line user to indicate where the burn is prior to segmentation, as it has been done in the past^24,25^ might improve the results; yet, it is doubtful that this procedure would be viable in real life settings.

Including images without burns in the data revealed a propensity for the algorithm to identify small burn areas where there were none in as much as a fifth of the images in the validation set. A slightly larger proportion of false positives was also reported in a previous deep-learning study^23^. Although images without any burn wounds are unlikely to be submitted by a front-line healthcare professional for diagnostic assistance, false positives could later on have consequences if the algorithm is used in sequence with a wound classifier algorithm in a fully automated approach. This could be the focus of future studies whereby training and testing with non-burn images is an integral part of the research question, and with the hypothesis that non-burn images could be provided to such a model by a clinician. In fact, previous studies have already studied the distinction between burn and other types of skin wounds^26,27^. Focus of further research could also include replicating the obtained results using an open-source software.

With regards to burn severity classification, most of the algorithms published did not apply CNNs but rather, handcrafted features were used to train mathematical or Support Vector Machine (SVM) models^24,25,29^. Using those methods, two studies also discriminated between the need for surgery or not, and classified accurately about 80% of the images^24,29^. These results are comparable to ours for patients of North-Germanic origin which had similar (lighter) skin types as in those previous studies, but higher than what we obtained with all cases aggregated. Nonetheless, there has been more recent CNN models that have classified burn depth in several categories including normal skin, with promising results between 80 and 95%^28,30,31,42^. Given the differences in category definition, these results are difficult to compare to the ones we have obtained.

The algorithm was trained using a commercially available software which did not permit to describe the actual architecture of the CNN models. Nonetheless, we believe that with the parameter information provided in this publication it should be possible to replicate the experiment. If a researcher requires access to the exact same algorithm, the corresponding author can be contacted for inquiries. Further, this study did not investigate other types of approaches to analyse the data such as SVM or decision trees. Differences in performances between approaches was not the aim of the study, and our choice of CNNs was based on previous literature where deep-learning algorithms outperformed other cited methods for image segmentation and classification^43^.

The depth of the burn correlates with the time to healing and scarring (and need for surgical intervention) and has therefore consequences on the clinical protocol. However, as indicated earlier, clinical evaluation of the depth is a challenging task. Our results suggest we could reduce this error to one in four cases among the lighter skin types but likely not among the darker ones. Image-based assessment of whether surgery is indicated or not has been tested on physicians of a range of competences, including referring physicians with a higher specificity (76.2%) but a much lower sensitivity than our algorithm (39.9% versus 92.5% respectively)^44^. It is also of note that in order to give an appropriate diagnosis, burn wounds should be cleaned and undressed. This is the case not only for clinical bed-side evaluations, but also for remote consultation. Therefore, we have only included such type of wound images in our dataset. It is possible that in the future less stringent criteria can be applied, and for an algorithm to be trained on uncleaned wounds, nonetheless, this will require some more complex data collection, annotation and model training.

Contrary to what was suggested previously by Abubakar and colleagues who also investigated two skin types, performing separate model trainings did not improve the results compared to the hybrid algorithm with regards to the AUC^26^. They however suggested that providing skin-type information as input to the algorithms would improve the results, which might also be the case in our algorithm since we find differences in results between skin types. It is also possible that all datasets collected to date are too small for this type of complexity level. It is nonetheless important to include patients of various skin types, and especially those of darker skin types as they are those where burn injuries are most prevalent^2^ and where care is the least available^15^.

The main strength of this study is that it is based on a large dataset with patient images diversified as regards type of skin, setting (with several centres from South Africa and Sweden), injured population (adults versus children), injured body parts, and burn mechanisms. An additional quality of the dataset is that it consists of images from real-life settings, acquired using various cameras and smartphones, in the same way they would be sent to a specialist for assessment.

A drawback of the dataset is that the classification as to whether surgery was needed or not was image-based rather than on specific areas of the images. The training was therefore weakly supervised, a procedure that will introduce noise in the labels and thereafter a loss in accuracy as the algorithm returns a value for each pixel. It is however also a strength in that it is a better representation of how a real-life remote consultation would proceed with a burn specialist. Both algorithms were analysed and presented separately in this study, it is however possible to envision that in the future they will form part of a single assessment, with the regions identified would then be fed for severity classification. Yet, this would require detailed pixel-level annotation even for the severity classification algorithm.

An additional issue is the differences between the material collected from both settings. The images from South Africa surpassed in number the ones from Sweden and contained most of the cases requiring surgery. Furthermore, their origin and quality were more heterogeneous as several burns centres and their referrals were included. These differences may have resulted in a slight overtraining towards the dark-skinned cases. However, the results of the wound classifier algorithm with much higher accuracy for images captured in Sweden shows this is probably not the case.

Among the annotations, whether surgery was required or not was established by a burn expert, a method that can lead to discrepancies between physicians^8^, but which was measured previously on a similar dataset with acceptable results^17^. Given the high number of images and the retrospective use of clinical diagnosis, difference between annotators could unfortunately not be measured in this study. A potential alternative would have been to wait for 21 days for wound closure, which would be particularly relevant given the dynamic nature of the burn wound in the first hours following a burn. This is however not feasible in settings where hospital beds are extremely limited and risk of infection is high^9^. Furthermore, additional variables such as the mechanism of the injury, or physical parameters such as blanching or capillary refill might have improved the surgical classification algorithm’s performances. On a similar note, in the first few days following the injury, the borders of the superficial burn areas can be difficult to define. Training of the annotators as well as close collaboration with the burn experts were intended to reduce the error in area definitions, nonetheless annotation errors cannot be excluded. However, given the high number of pixels included in a burn image and the fact that the majority of the pixels within the drawn polygon are likely to represent the true burn wound, this should not have affected the results in a significant manner.

Results in other disciplines such as dermatology, or ophthalmology have highlighted the potential added value of deep learning algorithms for improved diagnosis^45,46^. This study is a first step towards the development of such a system in burns care where, globally, the number of cases far exceeds the number of specialists available for assistance. To augment the accuracy of the algorithms, there might be a need for training in more homogeneous and refined subsets of data. However, these subsets might come short of being a good reflection in real-life settings. Additional challenges ahead would relate to the acceptance and trust of procedures of the like among all users , their seamless integration into health care services^47^, and the full respect of crucial ethical principles like patient autonomy and safety^48^.

From the front-line clinicians’ perspective, the accuracy results obtained are comparable to bedside levels of assessment, thus the algorithm could assist with their management of burns patients, relieving most of the stress usually observed. From the burns specialist’s perspective, a high sensitivity would minimize the risk of missing a burn that would require surgery, enhancing the probability of a good outcome for the patient as it would provide an accurate diagnosis while still reducing the number of referrals to a burns specialist who cannot attend all cases.

## Conclusion

This article provides evidence that two steps critical to burn assessment can be supported by image-based deep-learning algorithms among various skin types with high levels of accuracy for wound identification but also for wound classification with respect to the need for surgery, especially in lighter skin types. For an automated diagnosis to become a viable option to be used by frontline clinicians at point-of-care, the performance of deep learning algorithms must be re-trained and assessed on extended datasets representing even more variable clinical settings. Training using pixel-level annotations, identification and segmentation of burn depths could also be beneficial from a clinical standpoint in order to have a more precise diagnosis. Furthermore, for such systems to be implemented, acceptability and usability issues will have to be looked into.

## Supplementary Information

Supplementary Figure S1.

Supplementary Figure S2.

Supplementary Figure S3.

## Data Availability

The data that support the findings of this study were used under a license for the current study, and some restrictions apply to their availability. The data are available from the authors upon reasonable request.
