# Author Correction: Development and evaluation of deep learning algorithms for assessment of acute burns and the need for surgery

**DOI:** 10.1038/s41598-023-31508-9

**Published:** 2023-03-27

**Authors:** Constance Boissin, Lucie Laflamme, Jian Fransén, Mikael Lundin, Fredrik Huss, Lee Wallis, Nikki Allorto, Johan Lundin

**Affiliations:** 1 Department of Global Public Health, Karolinska Institutet, Stockholm, Sweden; 2 Department of Medical Epidemiology and Biostatistics, Karolinska Institutet, Stockholm, Sweden; 3 Institute for Social and Health Sciences, University of South Africa, Johannesburg, South Africa; 4 Department of Plastic and Maxillofacial Surgery, Burn Center, Uppsala University Hospital, Uppsala, Sweden; 5 Department of Surgical Sciences, Plastic Surgery, Uppsala University, Uppsala, Sweden; 6 Institute for Molecular Medicine Finland FIMM, Helsinki Institute for Life Science HiLIFE, University of Helsinki, Helsinki, Finland; 7 Division of Emergency Medicine, Faculty of Medicine and Health Sciences, Stellenbosch University, Bellville, South Africa; 8 Division of Emergency Medicine, University of Cape Town, Cape Town, South Africa; 9 Pietermaritzburg Burn Service, Department of General Surgery, University of Kwa-Zulu Natal, Pietermaritzburg, South Africa

Correction to: *Scientific Reports* https://doi.org/10.1038/s41598-023-28164-4, published online 31 January 2023

The original version of this Article contained errors. The names of the authors Jian Fransén, Fredrik Huss, Lee Wallis, Nikki Allorto and Johan Lundin were incorrectly given as Fransén Jian, Huss Fredrik, Wallis Lee, Allorto Nikki and Lundin Johan.

The original Article has been corrected.

