# Supplementary material for: Development and evaluation of deep learning algorithms for assessment of acute burns and the need for surgery
Source: Sci Rep. 2023 Jan 31;13:1794. doi: 10.1038/s41598-023-28164-4 (PMC9889389; doi:10.1038/s41598-023-28164-4)
Supplement: Supplementary file 1 — Supplementary Figure S1. [file 41598_2023_28164_MOESM1_ESM.pdf]

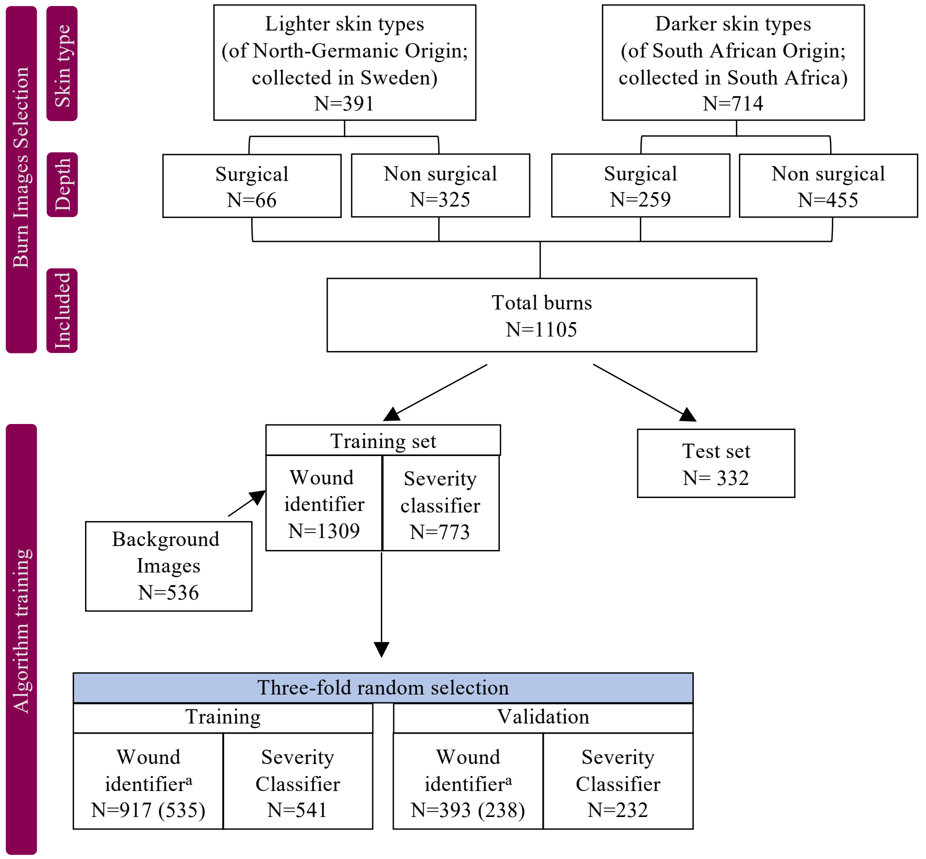

Figure S1. Consort diagram of included images in each of the algorithms

[a] For the wound identifier algorithms, analyses were performed both with and without background images. The number in parentheses represents the analyses with only burn images
