# Supplementary material for: Development and evaluation of deep learning algorithms for assessment of acute burns and the need for surgery
Source: Sci Rep. 2023 Jan 31;13:1794. doi: 10.1038/s41598-023-28164-4 (PMC9889389; doi:10.1038/s41598-023-28164-4)
Supplement: Supplementary file 2 — Supplementary Figure S2. [file 41598_2023_28164_MOESM2_ESM.pdf]

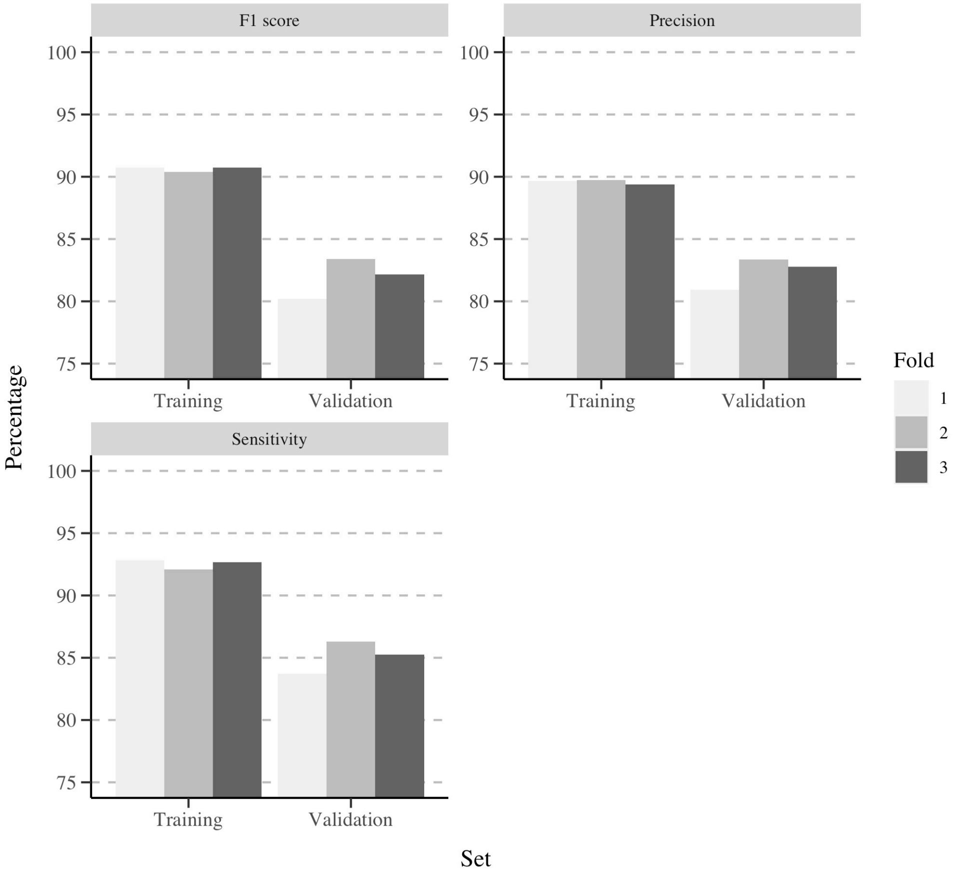

Figure S2. F1 score, Precision and Sensitivity results for each of the training and validation sets in the three-fold random selection of images
