# Supplementary material for: Development and evaluation of deep learning algorithms for assessment of acute burns and the need for surgery
Source: Sci Rep. 2023 Jan 31;13:1794. doi: 10.1038/s41598-023-28164-4 (PMC9889389; doi:10.1038/s41598-023-28164-4)
Supplement: Supplementary file 3 — Supplementary Figure S3. [file 41598_2023_28164_MOESM3_ESM.pdf]

**Wound area annotated**<br>Presented as a red line surrounding the wound	**Wound area identified**<br>Presented as the opaque red area (dark red line is area annotated)

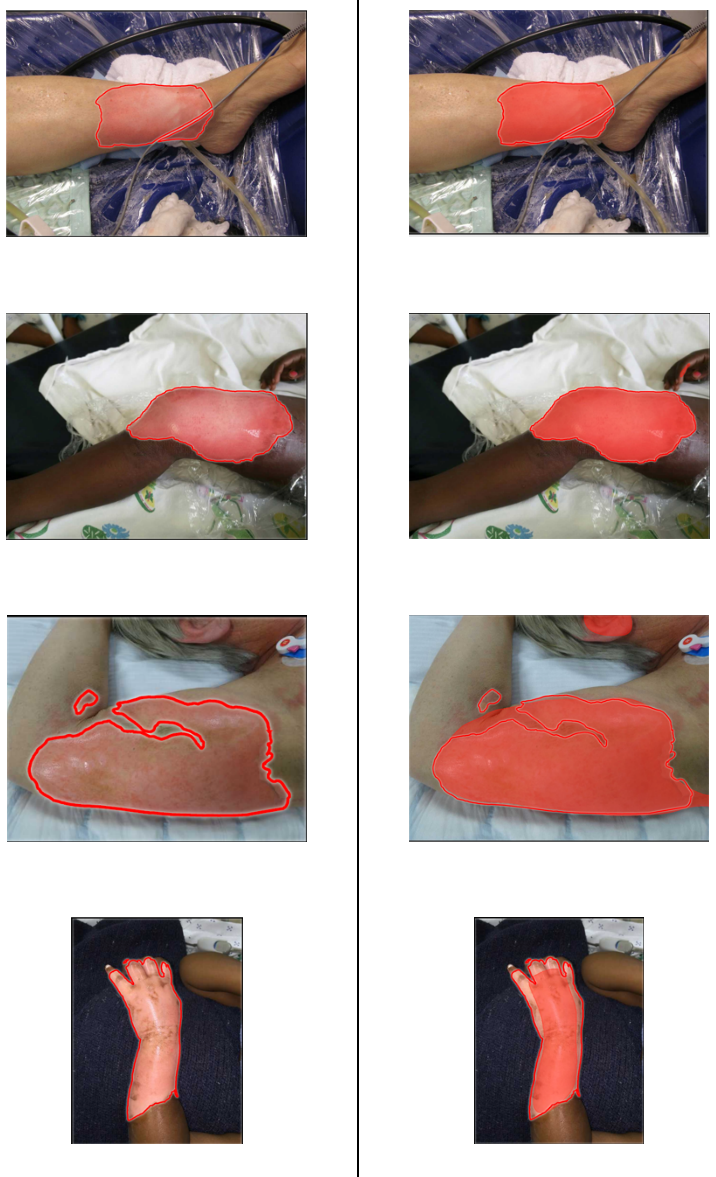

Figure S3. Examples of areas identified and segmented by the wound identifier algorithm. The dark red line represents the area annotated, while the opaque red area represents the area identified.
